# Association of vitamin D receptor gene polymorphisms with pancreatic cancer: A pilot study in a North China Population

**DOI:** 10.3892/ol.2013.1215

**Published:** 2013-02-28

**Authors:** LEI LI, BO WU, LIBO YANG, GUANCHENG YIN, WEI WEI, SHUJING SUI, JIYONG LIU

**Affiliations:** 1 Department of Gastroenterology, Shandong Provincial Hospital, Shandong University, Jinan 250021;; 2 Department of Internal Medicine, Taian City Central Hospital, Taian 271000, P.R. China; 3 Department of General Surgery, Taian Eighty-Eight Hospital, Taian 271000, P.R. China

**Keywords:** vitamin D receptor gene, *Fok*I, *Bsm*I, polymorphism, pancreatic cancer, North China

## Abstract

Polymorphisms of the vitamin D receptor (VDR) gene may be a risk factor for pancreatic cancer (PC). We investigated the association of two single-nucleotide polymorphisms (SNPs) of the VDR gene with PC in age- and gender-matched patients and controls. PC (n=91) and healthy control (n=80) samples were genotyped for the *Fok*I (rs2228570) and *Bsm*I (rs1544410) polymorphisms using the PCR and restriction fragment length polymorphism (PCR-RFLP) method. Chi-square analysis was used to test for the overall association of VDR genotype with disease. There was a significant difference in the frequency of genotype FF between the PC patients and controls (P^trend^=0.009); however, the difference in frequency of genotype BB between the two groups was not significant (P^trend^=0.082). The difference between FF and Ff/ff frequency was significant (P=0.002). The two high-risk genotypes were ffbb and Ffbb, with an 11.66- and 6.42-fold increased risk of PC, respectively. VDR gene polymorphisms were important for the development of PC in this study population; however, further exploration of these findings and their implications are required.

## Introduction

Pancreatic cancer (PC) is one of the most lethal human malignant tumors and accounts for 3% of all reported cases of cancer (1). It is estimated to have been responsible for >250,000 mortalities and was the fifth leading cause of cancer-associated deaths worldwide in 2007 (2). The prognosis for PC is extremely poor, with a 5-year survival rate of <5%, even with surgical and chemotherapeutic intervention (3). It has been shown that 1α,25(OH)_2_D_3_ acts as a type of hormone and significantly inhibits the proliferative activity of numerous types of cancer cells, including PC cells, *in vitro* and regulates growth and differentiation in various cell types (4,5). It acts by binding to a corresponding intranuclear vitamin D receptor (VDR), which is present in a number of target tissues (6,7).

Numerous studies have demonstrated that polymorphisms of the VDR gene have important implications in VD signaling and are associated with various malignancies, including cancer of the colon, breast, kidneys and prostate (8–12). However, little is known about the role of the VD endocrine system in the carcinogenesis of PC.

Therefore, the aim of this study was to screen for genetic variations of two single-nucleotide polymorphisms (SNPs), *Fok*I (rs2228570) and *Bsm*I (rs1544410)*,* of the VDR gene in a well-defined population of individuals with PC and compare their incidence with that of a healthy control population, in order to determine the contribution of VDR polymorphisms to PC in North China.

## Materials and methods

### Study participants

This study was part of an ongoing hospital-based case-controlled study, conducted at three hospitals (Shandong Provincial Hospital, Taian Central Hospital and Taian Eighty-eight Hospital, Shandong, China). The purpose of this study was to define risk factors which contribute to the development of PC. PC patients (n=91) eligible for the current study were enrolled between January 1 2010 and June 30 2012. Diagnosis of the samples was confirmed by certified histopathologists (only pancreatic adenocarcinoma was included) in the three hospitals mentioned above. Formalin-fixed paraffin-embedded tissues from PC patients (n=91) were used. The control group consisted of 80 healthy volunteers, selected by age- and gender-matching to the PC patients. The volunteers had no history of any type of cancer at the time of recruitment. Written informed consent was obtained from patient’s and volunteer’s families. This study was approved by the Ethical Committees of the three hospitals.

### DNA isolation in PC

The required thin (10×10 *μ*m) tissue sections were dried onto slides at 37°C overnight. After soaking the tissue sections in xylene, deparaffinization was carried out with ethanol series for 3–5 min each (100% ethanol for dehydration and 80, 60 and 40% ethanol). To differentiate healthy tissue from tumor tissue, the slides were stained with hematoxylin. Tumor tissues were isolated by microdissection and the DNA was extracted using a DNA Isolation kit (Sangon Biotech, Inc., Shanghai, China), according to the manufacturer’s instructions.

### DNA isolation from peripheral venous blood of control population

Peripheral venous blood was obtained from each healthy volunteer and promptly centrifuged (1,500 × g for 10 min). Genomic DNA was extracted from 200 *μ*l EDTA blood with a DNA Isolation kit from Roche Diagnostics (Sangon Biotech, Inc.), according to the manufacturer’s instructions. To obtain higher DNA concentrations, a number of blood samples intially underwent lymphocyte separation, performed according to the manufacturer’s instructions (Sangon Biotech, Inc.). Briefly, 3 ml diluted blood samples were carefully centrifuged at 1,200 × g for 20 min at 25°C and lymphocytes from the interphase were washed twice in phosphate-buffered saline (PBS). DNA was then isolated as described above.

### Genotyping of FokI

According to the method described by Arjumand *et al*(11) and Harries *et al*(13), the polymorphisms of VDR [*Fok*I (rs10735810) and *Bsm*I (rs1544410)] were assayed using the PCR and restriction fragment length polymorphism (PCR-RFLP) method. Genomic DNA (2 *μ*l) was used, in addition to 200 ng of forward and reverse primers, 1X *Taq* polymerase buffer (1.5 M MgCl2), dNTPs (0.3 mM) and 1 unit of *Taq* DNA polymerase (Sangon Biotech, Inc.). The primers of the VDR gene used were forward (5′-AGCTGGCCCTGGCACTGACTCTGCTCT-3′) and reverse (5′-ATGGAAACACCTTGCTTCTTCTCCCT-C-3′). PCR amplification was carried out with the following cycling parameters: denaturation at 94°C for 5 min, 35 cycles at 94°C for 30 sec, 61°C for 30 sec and 72°C for 1 min and one final cycle of extension at 72°C for 7 min. The C/T polymorphism in the first of the two-start codon (ATG) at the translation initiation site of the VDR gene was detected by RFLP, using the restriction endonuclease *Fok*I (Sangon Biotech, Inc.). The PCR product of the 265-bp band was digested with 5 units of *Fok*I restriction enzyme and incubated at 37°C for 4 h. The digested reaction mixture (10 *μ*l) was then loaded using 2% agarose gel containing ethidium bromide and visualized under short-wave UV light. The sizes were determined using a 100-bp ladder (Sangon Biotech, Inc.). Digestion of the amplified 265-bp PCR product yielded two fragments: 169 and 96 bp. Depending on the digestion pattern, individuals were scored as FF when homozygous for the presence of the *Fok*I site, ff when homozygous for absence of the *Fok*I site or Ff in the case of heterozygosity.

### Genotyping of BsmI

The PCR amplification was carried out with the following cycling parameters: denaturation at 94°C for 5 min and 35 cycles at an annealing temperature of 66°C with the following primers: forward (5′-CAACCAAGACTACAAGTACCGCGTCAGTGA-3′) and reverse (5′-AACCAGCGGGAAGAGGTCAAGGG-3′). The 800-bp PCR product was then diluted and digested with enzyme *Bsm*I at 65°C for 18 h using 5 units of enzyme (Sangon Biotech, Inc.) for each 20 *μ*l reaction. After digestion, the PCR products were separated using 2% agarose gel containing ethidium bromide and visualized under short-wave UV light. Fragments of 650 and 150 bp were visible after the 800-bp product was digested by the *Bsm*I restriction enzyme. DNA from homozygotic individuals lacking a *Bsm*I restriction site (BB) appeared on the gel as a single 800-bp band. All the primers were synthesized by Sangon Biotech, Inc.

### Statistical analysis

Statistical analysis was performed using SPSS 13.0 statistical software (SPSS Inc., Chicago, IL, USA) and data are presented as mean ± SD. Comparisons between two groups were performed using independent t-tests. The χ^2^ analysis was applied to determine the difference in the genotype and gene frequency. Odds ratios (ORs) and 95% confidence intervals (95% CIs) were calculated from unconditional logistic regression models. P<0.05 was considered to indicate a statistically significant difference.

## Results

### Patient characteristics

In our study, 91 PC patients (52 males and 39 females) were diagnosed histopathologically following surgery or endoscopic ultrasonography fine-needle aspiration (EUS-FNA) and the mean age of patients was 47.1±9.1 years. We recruited 80 healthy volunteers (controls; 45 males and 35 females) and their mean age was 47.5±7.4 years (Table I). Initially, Pearson’s χ^2^ test was performed to examine the genotypic distribution of the control population. VDR F*ok*I and B*sm*I genotypic distributions were calculated according to the Hardy-Weinberg equilibrium, with P-values of 0.156 and 0.606, respectively (Table II). The PC patients and control population were age- and gender-matched using Chi-square analysis, revealing P-values of 0.718 and 0.906, respectively, as shown in Table I.

### VDR FokI polymorphism

As shown in Table III, the number of PC patients with genotype FF was 22 (24.2%), Ff was 46 (50.5%) and ff was 23 (25.3%). For the control population, there were 37 (46.3%), 31 (38.7%) and 12 (15.0%) cases with FF, Ff and ff, respectively. Thus, the difference in the occurrence of the FF genotype between the PC patients and control population was significant (P^trend^=0.009). The percentage of PC patients with genotypes FF or combined Ff/ff was 24.2% and 75.8% and for the control population was 46.3% and 53.7%, respectively. This difference was statistically significant (P=0.002; OR=2.699; 95% CI, 1.408–5.173). There was a significant difference between genotypes FF and Ff (P=0.009; OR=2.496; 95% CI, 1.243–5.011) and genotypes FF and ff (P=0.008, OR=3.223, 95% CI, 1.344–7.733). The incidence of F and f alleles for the *Fok*I polymorphism was 90 (49.4%) and 92 (50.6%) cases in the PC patients and 105 (65.6%) and 55 (34.4%) cases in the control population, respectively. The difference in the allele frequency between the two groups was statistically significant (F vs. f allele; P=0.035; OR=1.952; 95% CI, 1.261–3.021).

### VDR BsmI polymorphism

As shown in Table III, the frequencies of the BB, Bb and bb genotypes in PC patients were 23 (25.3%), 40 (44.0%) and 28 (30.7%) and in the control population were 26 (32.5%), 41 (51.2%) and 13 (16.3%), respectively. There was not a significant difference in BB frequency between the PC and control populations (P^trend^=0.082). The genotype frequency percentage of BB and combined Bb/bb was not significantly different (P=0.297; OR=1.424; 95% CI, 0.732–2.768) between the PC patients (25.3 and 74.7%, respectively) and control population (32.5 and 67.5%, respectively); however, there was a significant difference between genotypes BB and bb (P=0.042; OR=2.435; 95% CI, 1.026–5.780). The frequency of B and b alleles for the *Bsm*I polymorphism were 86 (47.3%) and 96 (52.7%) in PC patients and 93 (58.1%) and 67 (41.9%) in the control population, respectively. The difference between the two groups was statistically significant (B vs. b allele; P=0.045; OR=0.645; 95% CI, 0.421–0.990).

### Combined analysis of genotypes FokI and BsmI

We pooled the data for *Fok*I and *Bsm*I genotypes of the VDR gene for PC patients and the control population to analyze the cumulative effect of *Fok*I and *Bsm*I polymorphisms, as shown in Table IV. Individuals with genotype ffbb had an 11.66-fold risk of PC compared with those of genotype FFBB (OR=11.667; P=0.009) and genotype Ffbb individuals had a 6.417-fold risk of PC compared with those of genotype FFBB (OR=6.417; P=0.018). There were no significant differences in risk of PC between the other genotypes, as shown in Table IV.

## Discussion

PC is one of the most lethal types of human cancer, responsible for >250,000 mortalities and the fifth leading cause of cancer-associated deaths worldwide in 2007 (2). The majority of patients who contract the disease usually succumb to it within a few months of diagnosis, despite surgical or medical intervention. The incidence rate of PC is approximately the same as the mortality rate and the 5-year survival rate is <1% (14). Carcinoma of the exocrine pancreas is an increasingly common cancer, but no effective chemotherapy has been developed for patients with advanced disease (15). It has previously appeared that receptors in PC, such as estrogen receptors (ER), may be responsive to endocrine therapy. However, subsequent clinical trials with these receptors have not supported this therapy (16).

Epidemiological studies show that individuals living at higher latitudes are at an increased risk of PC and are more likely to succumb to the cancer than those living at lower latitudes (5). One reason is that 1,25(OH)_2_VitD affects >200 genes to regulate proliferation, differentiation, apoptosis and angiogenesis of cells (17–19). Also, *in vitro* studies have demonstrated that 1,25(OH)_2_VitD and its synthetic analogs are able to inhibit the proliferation of PC cell lines (20). The VDR and its gene polymorphisms may also be important (21). Genetic studies have investigated the possible association between various histotypes of cancer and the detection of specific SNPs of the VDR gene (VDR-SNPs). Polymorphisms in the VDR gene have been shown to affect VDR mRNA and protein levels (22), which in turn may affect the immunomodulatory function of VDR (23). Approximately 200 different VDR-SNPs have been described; however, the VDR polymorphisms which are most frequently associated with tumorigenesis are *Fok*I, *Bsm*I, *Taq*I, *Apa*I, *Eco*RV and Cdx2 (24–26). The most frequently studied SNPs are the RFLPs *Fok*I and *Bsm*I. The *Fok*I RFLP, located in the coding region of the VDR gene, leads to the production of a VDR protein that is three amino acids longer than normal. Although no significant differences in ligand affinity, DNA binding or transactivation activity have been identified between these two VDR forms, the shorter VDR variant exhibits higher potency than the longer one. The *Bsm*I RFLP is intronic and located at the 3′ end of the gene. *Bsm*I does not alter the amount, structure or function of the final VDR protein produced, but it is strongly linked with a poly(A) repeat and may affect VDR mRNA stability. Thus, VDR polymorphisms have important implications for VD signaling and are associated with various malignancies (27,28). A number of studies have reported the role of the VDR gene in different malignancies, yet no studies have been carried out to evaluate the role of VDR gene polymorphisms in PC. To the best of our knowledge, this is the first study to investigate the association between VDR polymorphisms and the risk of PC.

VD is involved in the regulation of cell proliferation and differentiation *in vitro* and *in vivo*(29). The results of this study revealed that genetic heterozygous variants of *Fok*I are associated with a decreased risk of PC in a North Chinese population, whereas the effects of *Bsm*I were not significant. The difference in the allele frequencies was statistically significant (F vs. f allele, P=0.003; OR=1.952; 95% CI, 1.261–3.021. B vs. b allele, P=0.045; OR=0.645; 95% CI, 0.421–0.990) between PC patients and the control population. When the two genotypes, *Fok*I and *Bsm*I*,* were combined to analyze their cumulative effect in PC, we identified that ffbb and Ffbb genotypes have an increased risk of PC. This may be attributed to the haplotypic effect associated with the linkage disequilibrium of these two polymorphic sites. Thus, we propose that the *Fok*I and *Bsm*I polymorphisms of VDR are potential prognostic variables which may predict the risk of developing PC in the North Chinese population.

A number of potential improvements may be considered for any further studies. Firstly, more PC and control samples are required to effectively test the effects of variables. Secondly, further investigation of VDR-SNPs, including *Taq*I and *Apa*I, to analyze the association between SNPs and PC and various clinicopathological parameters for PC patients and healthy volunteers is required. Comparison of the various risk factors for PC (age, smoking, diabetes and chronic hepatitis B infection) and the frequency of VDR genotypes in PC patients may also be considered for further investigation.

In conclusion, data from this study showed that the *Fok*I and *Bsm*I polymorphisms were associated with a higher risk of PC among the North Chinese population. Furthermore, this study showed for the first time that these two polymorphisms in the VDR gene are potential determinants in PC patients. The main aim of this study was to understand the role of VDR gene polymorphisms in the etiology of PC in China. Additional studies on a larger population size are warranted to elucidate the role of genetic variations of VDR and PC risk.

## Figures and Tables

**Table I:** Characteristics of the study population.

Group	Male, n	Female, n	Age (years, mean ± SD)
PC	52	39	47.1±9.1
Control	45	35	47.5±7.4

PC, pancreatic cancer. Age, P=0.718; Gender, P=0.906.

**Table II:** Genotype distribution in the Hardy-Weinberg equilibrium.

Genotype	Predictive value (%)	Observed value (%)	χ^2^	P-value
*Fok*I			2.015	0.156
CC	43.1	46.3		
CT	45.1	38.7		
TT	11.8	15.0		
*Bsm*I			0.266	0.606
CC	33.8	32.5		
CT	48.7	51.2		
TT	17.5	16.3		

**Table III:** Association of VDR *Fok*I and *Bsm*I polymorphisms and PC risk.

	Group				
Genotype	PC, n (%)	Control, n (%)	OR	95% CI	P-value
*Fok*I^a^					
FF (CC)	22 (24.2)	37 (46.3)	1		
Ff (CT)	46 (50.5)	31 (38.7)	2.496	1.243–5.011	0.009
ff (TT)	23 (25.3)	12 (15.0)	3.223	1.344–7.733	0.008
Ff+ff	69	43	2.699	1.408–5.173	0.002
FF	22	37			
FF+Ff	68	43			
F	90 (49.5)	105 (65.6)	1		
f	92 (50.5)	55 (34.4)	1.952	1.261–3.021	0.003
*Bsm*I^b^					
BB (AA)	23 (25.3)	26 (32.5)	1		
Bb (AG)	40 (44.0)	41 (51.2)	1.103	0.542–2.244	0.787
bb (GG)	28 (30.7)	13 (16.3)	2.435	1.026–5.780	0.042
Bb+bb	68	54	1.424	0.732–2.768	0.297
BB	23	26			
Bb+bb	68	54			
B	86 (47.3)	93 (58.1)	1		
b	96 (52.7)	67 (41.9)	0.645	0.421–0.990	0.045

VDR, vitamin D receptor; PC, pancreatic cancer; OR, odds ratio; CI, confidence interval.

a P^trend^=0.009;

b P^trend^=0.0082.

**Table IV:** Combined analysis of VDR *Fok*I and *Bsm*I genotypes between PC patients and controls.

	Group				
Genotype	PC, n (%) Total n=91	Control, n (%) Total n=80	OR	95% CI	P-value
FFBB	6 (6.59)	14 (17.50)	1		
FFBb	9 (9.89)	16 (20.00)	1.313	0.373–4.616	0.671
FFbb	7 (7.69)	7 (8.75)	2.333	0.565–9.639	0.296
FfBB	10 (10.99)	8 (10.00)	2.917	0.768–11.070	0.188
FfBb	25 (27.47)	19 (23.75)	3.07	0.995–9.477	0.062
Ffbb	11 (12.09)	4 (5.00)	6.417	1.444–28.511	0.018
ffBB	7 (7.69)	4 (5.00)	4.083	0.861–19.371	0.128
ffBb	6 (6.59)	6 (7.50)	2.333	0.53–10.267	0.288
ffbb	10 (10.99)	2 (2.50)	11.667	1.940–70.178	0.009

VDR, vitamin D receptor; PC, pancreatic cancer; OR, odds ratio; CI, confidence interval.
